# The Teething Myth
1A paper read at a Meeting of the Bristol Medico-Chirurgical Society on Wednesday, 9th March, 1921.


**Published:** 1921-06

**Authors:** R. C. Clarke

**Affiliations:** Assistant Physician to the Bristol Royal Infirmary


### THE TEETHING MYTH.[1]

BY

R. C. CLARKE, O.B.E., M.B., B.Ch. Brist., M.R.C.P.,

*Assistant Physician to the Bristol Royal Infirmary.*

TEETHING is a matter which looms largely on the horizon of those who have the care of infants, be it in the nursery at home, in private practice, in hospital, or at the various Welfare Centres which are springing up to-day.

Teething is a subject with a long history. Hippocrates stated that at the approach of dentition itching of the gums, fever, convulsions and diarrhœa occurred, especially in those who were fat or had constipated bowels. Most

[1] A paper read at a Meeting of the Bristol Medico-Chirurgical Society on Wednesday, 9th March, 1921.

of the ancient writers deal with the subject in much the same tone, though the diversity of opinion as to which were the most difficult teeth and which was the worst season of the year lead one to suppose that they were not very sure of their ground. Some blame the incisors as they are the sharpest, others the molars as they are the bluntest.

In the sixteenth, seventeenth and eighteenth centuries every conceivable ailment of infants was attributed to this cause. The edentulous period, that is, the first six months of life, did not defeat these enthusiasts, as they called this the period of the "breeding of the teeth." Very divergent views were held as to the actual cause of the trouble, though the main one was the keeping back of the tooth by a hard and resisting gum. A large number of methods for softening the gum were devised, which varied from the simple process of rubbing the gums with butter or decoctions of herbs to the more elaborate remedies, such as sucking pig's brains, the milk of a bitch, or blood from a freshly-killed cock's comb. Many weird ideas sprang up at this time, some of which survive to this day, a particularly pernicious one being that an ear should never be allowed to stop discharging till the last tooth is cut.

From time immemorial mothers and doctors seem to have had the idea that nature must be helped to break the gum, and many and varied are the implements that have been recommended. A wolf's tooth was at one time popular, and, worse to relate, a finger-nail always had its vogue. This practice survives to this day. Ambrose Paré was the first surgeon to advocate the opening of the gums with a lancet. It came about in this way. He and the court physicians were hunting about for the cause of death in a child aged eight months, the son of the Duc de Nevers, and they found nothing till he opened the gums and discovered, in his own words, "all his teeth as it were in array ready to come out, which, had it been done when he lived, doubtless he would have been saved." Paré performed this operation on all his own children, though he does not give the mortality. Opening the gums as a surgical operation seems to have become fashionable in England in the early part of the eighteenth century.

In the early part of last century dentition as a cause of disease looms largely in the text-books, and half the infant mortality was attributed to it. In the middle of the century one finds doubts creeping in, earlier in the French than in the English books. In the Registrar-General's reports dentition is progressively weakening as a cause of death. In 1904 there were just under 3,000 deaths certified from this cause, while in 1918 1,011 was the total.

In the modern text-books the subject is sometimes dealt with by the simple process of hedging. In one system on children's diseases the subject is ignored as though it were indecent. Dr. Still seems to be a confirmed believer in the evils of teething, his main argument being that he cannot believe that so many generations of intelligent parents can have been deceived, an argument which is most fallacious. It is no longer the custom to protect children from the evil eye, from being overlooked, or from a "sending," yet countless generations of intelligent parents did this. No one sends scrofulous cases to be touched by His Britannic Majesty George V., yet, if we are to believe Macaulay, not only "intelligent parents," but the highest physicians and surgeons in the land believed in this practice for years. Charles II. spent considerable time in "touching," and William III. became most unpopular because he frankly disbelieved in it and strove to cut down their numbers. Many similar instances occur to one, and there is no need to labour the point.

One of the chief difficulties of arriving at a just estimation of facts lies in the unfortunate truth that no one knows the exact method by which a tooth is cut. The only thing we know is that in due course a tooth is either pushed up or moves up, and the gum atrophies over it. One thing is certain, and that is that the tooth does not bore its way through like a gimlet, which is the popular idea. Cutting teeth is probably not an essentially painful process. No one maintains that every tooth causes pain, and it is reasonable to suppose that if one is painful all would be. The second dentition, though now the erupting tooth causes atrophy of the fang or the milk tooth and frequently has to pierce the gum as well during eruption, is painless unless the milk tooth be carious. A wisdom tooth is not a fair test, as it frequently cannot get through owing to want of room in the gum. If there is plenty of room a wisdom tooth is cut painlessly, and many people are unaware of the process.

There is yet another pitfall. A baby starts cutting its teeth at between four and ten months old, and the process continues until the end of the second year. Sometimes the teeth appear rapidly, sometimes there is a long pause. It is quite impossible to say, in a given case, when a tooth is seen to bulge the gum, how long it will be before the tooth will pass the time-honoured test of ringing on a metal spoon. One tooth will appear before its impending arrival is suspected while another will bulge the gum for as much as a month before it shows through. In fact, it comes to this, that if one looks into any baby's mouth from four months to two years old it is possible to find that a tooth is either bulging the gum, just coming through, or has actually just come through. Therefore one who believes in the evil of teething can always find an excuse for an illness in a baby's mouth.

The so-called signs and symptoms of teething may be divided into two classes, the mild or physiological ones (being about his teeth) and the difficult or pathological ones (cutting his teeth hard).

Now to go through these signs and offer an alternative cause in each. To take the physiological ones first.

*Dribbling.*—A normal baby starts dribbling at about three or four months old, due to the functions of the salivary glands developing. This goes on till the infant is a year old, when it probably learns to swallow its saliva like a reasonable member of a civilised community. As it starts before the eruption of the teeth and ends long before the completion of the process, surely dentition can have nothing to do with dribbling.

*Biting on dummies and hard rings.*—Anyone who has watched a baby develop must have observed that as a function develops the instinct to practise it develops at the same time. With the eruption of the teeth the instinct to bite appears, so that a baby will bite anything that is put into its mouth. This fact is no more evidence of itching gums than a baby which sits up patting the table is of itching fingers.

*Putting objects into the mouth.*—The principal impressions an infant obtains depend on its relation to food and drink; obtaining nourishment is the largest thing in its consciousness, and experience teaches it that all the best things in life go into its mouth. The lips also are used as a means of touching and feeling the quality of objects. In the twelfth week the movements of the hands become volitional, and what more natural than to use this feat by putting objects into its mouth.

*Being cross and perverse.*—In this matter babies differ little from their parents—they have their good days and their bad days. This variability of temper, as one would expect, becomes more marked as the mind develops. It is a comforting though fallacious doctrine for parents to attribute natural sin to teething.

*Rashes of various kinds.*—Red gum, which is a form of urticaria, is probably due to the ingestion of foreign protein. It is much more common in the first three months of life. Miliaria is due either to pyrexia or to too many clothes, but is invariably attributed to teething. Septic and irritative rashes are due either to infection or irritant forms of soaps and cosmetics.

*Sore gums and gingivitis.*—This is very uncommon, and is almost invariably caused by the pernicious practice of rubbing and irritating the gums with hard objects. Scurvy is another cause, though it is very uncommon in this district.

Now the pathological signs of dentition.

*Fever, restlessness, fretfulness and disturbed sleep.*—Surely it will be granted that these are the common manifestations of infant disorders from vaccination to concussion of the brain.

*Gastro-enteritis.*—Diarrhœa and vomiting is an extremely common ailment of infants, as common as teething itself, but the following points may be enunciated: (1) Bottle-fed babies are more liable to attacks than breast-fed ones in the first seven or eight months of life, the age they start to crawl, pick up dirty objects and put them into their mouths; (2) that the number of attacks of diarrhœa varies in inverse proportion to the care bestowed upon the baby; (3) that gastro-enteritis is infinitely less common in the class who do not use dummies or put dirty fingers and other objects into a baby's mouth; (4) that in Infant Clinics it fairly easy to guess which babies will "cut their teeth with diarrhœa" from the personal appearance of their mothers and the reports on the cleanliness of their homes by the visitor.

From the foregoing it may be inferred that infection is the paramount cause of gastro-enteritis in babies.

*Colds in the head.*—The younger the baby the more susceptible is it to nasal infection and to the disastrous spread of that infection to the middle ear and bronchial tubes. Colds, bronchitis and otitis media are far commoner in the first three months of life than during the period of dentition. Out of 40 infants under my care in the last two years who had bronchitis 32 definitely had their first attack before they were three months old. No one will disagree with the assertion that an infant that has once had bronchitis is more liable to subsequent attacks than one who has escaped. Therefore why, when a baby has had three attacks of bronchitis and two of otitis media in the period before dentition is every similar attack subsequently attributed to teething? Moreover, it is almost always those mothers with chronic nasal catarrh and its aural complications whose babies cut their teeth with a cold, bronchitis and discharging ears.

*Convulsions.*—Infants, owing to the surprising instability of their nervous system, are particularly prone to convulsions. It is impossible to deny that peripheral irritation may start a convulsion, though it is far more probable that a toxæmia is at the bottom of it. Convulsions are commoner in the first three months of life and in the edentulous rickety child.

It is not denied that the eruption of a tooth may be painful. Everyone is familiar with the peevish infant with dry, red, painful gums, pyrexia, sometimes alarmingly high and possibly in convulsions. Such a child, however, invariably has gastro-enteritis or bronchitis, or both. Why then is not the painful dentition secondary to the catarrhal disease? It is a platitude to state that the mouth is only a part of the gastro-intestinal tract; the tongue is a notoriously valuable guide to its condition. What more natural, then, when there is gastro-intestinal or respiratory infection, that the gums, where active change is taking place, should be involved in the general debacle? It is maintained, therefore, that painful dentition is a secondary complication and not a primary agent.

The whole field of medicine is full of so-called scientific theories to explain popular superstition. Some may be correct, the majority are incorrect, but all are equally plausible. Teething is no exception, and various theories have been given to account for the evils.

Of course there is the reflex theory. To make this a more plausible one it has been ordained that the fifth nerve supplies the teeth, a nerve which has communication with most of the cranial nerves, including the vagus, and with the sympathetic and para-sympathetic systems. The net is wide-spread. In one modern text-book there are beautiful diagrams showing the connection with the fifth nerve, through the otic ganglion to the vaso-motor nerve of the artery of the ear drum. This to explain otitis media in teething is most unconvincing. The fons et origo of this mysterious reflex is supposed to be the pressure of the base of a tooth on a nerve ending because it is being kept back by a resisting gum. Were this true it seems that pressure on a baby's gum, or the fact of a baby biting on a hard ring, would exaggerate the reflex, yet the same people affirm that the infant is relieved by this procedure.

Another theory, older and not so plausible as the former, is the pyrexia theory. Teething causes pyrexia, pyrexia causes chill, and chill accounts for everything. The obvious fallacy of this is that the eruption of a tooth does not *per se* cause pyrexia, as it is reasonable to suppose that if one did all would, a fact which no one would affirm. Moreover, chill has rather gone out of fashion of late years as a cause of disease.

The writer has discussed this subject with a certain number of people of experience, lay and medical. Among doctors there are two attitudes taken on the subject. There are those who believe in the evils of teething, and there are those who in their heart of hearts do not believe in it, but take the view that it is a very innocent and eminently useful diagnosis with which to appease an anxious mother. It is no use for the first class to read these lines. They learnt it at their mother's knee, and it is as firmly fixed in their minds as their religious or political beliefs. If one arose from the dead it would not convince them. It is to the second class that these remarks are addressed, for the following reasons. All the minor ailments of infants, with their disastrous sequelæ, are largely preventable and avoidable by careful management. Eruption of teeth, on the other hand, is an act of God and unpreventable. One gets better results if one tells Mrs. Smith of Pennywell Road that if she will persist in licking her baby's dummy after it is dropped before putting it back into his mouth, whereby he acquires the flora and fauna of the road and of her septic teeth at the same time, she must expect him to get diarrhœa, than if one orders a teething powder ; again, if one tells one's prosperous patient that if she will sack her adenoidy under-nurse there is a hope that her son and heir will not cut his next tooth with bronchitis. Furthermore, most babies will do better without the unprovoked assaults upon their insides in the form of teething powders. Lastly, there is rickets. There is no more easily or commonly overlooked disease than early rickets. This applies to rich and poor alike. These children are particularly disposed to the so-called teething troubles, attacks of pyrexia, convulsions and catarrhal infections of the respiratory and intestinal tracts, and it is this very " teething diagnosis " which puts practitioners on the wrong track, a really disastrous state of affairs, as rickets is a very easily cured disease.

